# Lorazepam during breastfeeding: a pharmacokinetic case report and exploratory pharmacovigilance assessment – a contribution from the ConcePTION project

**DOI:** 10.3389/fphar.2026.1877310

**Published:** 2026-06-24

**Authors:** Ghazaleh Shitreh, Martje Van Neste, Michael Ceulemans, Paola Mian, Nadir Yalçın, Hülya Tezel-Yalçın, Rasmus Huan Olsen, Christina Gade, Daan Touw, Pieter Annaert, Anne Smits, Karel Allegaert

**Affiliations:** 1 Clinical Pharmacology and Pharmacotherapy, Department of Pharmaceutical and Pharmacological Sciences, Leuven, Belgium; 2 Woman and Child, Department of Development and Regeneration, Leuven, Belgium; 3 Teratology Information Service, Netherlands Pharmacovigilance Centre Lareb, ‘sHertogenbosch, Netherlands; 4 Research Foundation Flanders (FWO), Brussels, Belgium; 5 Department of Clinical Pharmacy and Pharmacology, University Medical Center Groningen, University of Groningen, Groningen, Netherlands; 6 Department of Pediatrics, Beatrix Children’s Hospital, University Medical Center Groningen, University of Groningen, Groningen, Netherlands; 7 Department of Pharmaceutical Technology and Biopharmacy, Groningen Research Institute of Pharmacy, University of Groningen, Groningen, Netherlands; 8 Pharmacometrics Expertise Center of the Northern Netherlands, University Medical Center Groningen, University of Groningen, Groningen, Netherlands; 9 Department of Clinical Pharmacy, Faculty of Pharmacy, Hacettepe University, Ankara, Türkiye; 10 Department of Pharmaceutical Toxicology, Faculty of Pharmacy, Hacettepe University, Ankara, Türkiye; 11 Department of Clinical Pharmacology, Copenhagen University Hospital – Bispebjerg and Frederiksberg, Copenhagen, Denmark; 12 Department of Clinical Medicine, University of Copenhagen, Copenhagen, Denmark; 13 Drug Delivery and Disposition, Department of Pharmaceutical and Pharmacological Sciences, Leuven, Belgium; 14 BioNotus CommV, Niel, Belgium; 15 Neonatal Intensive Care Unit, University Hospitals Leuven, Leuven, Belgium; 16 Department of Hospital Pharmacy, Erasmus University Medical Center, Rotterdam, Netherlands

**Keywords:** breastfeeding, case report, human milk, lactation, lorazepam, pharmacokinetics, pharmacovigilance

## Abstract

**Introduction:**

Knowledge regarding lorazepam use during breastfeeding is limited and so are quantitative data on lorazepam transfer into human milk. Lorazepam transfer to human milk was characterized in a single case and pharmacovigilance databases were explored.

**Methods:**

A clinical lactation study was conducted in a breastfeeding mother who used lorazepam as needed for anxiety symptoms (single 1 mg oral dose, 6.5 months postpartum). Serial human milk (n = 6) and maternal plasma (n = 2) samples were collected over 24 h. Concentration-time data were used to calculate plasma and milk AUC_0–24h_ (linear trapezoidal interpolation), milk-to-plasma (M/P) ratios, daily and relative infant dose (DID, RID). Because of the *a priori* pragmatic design (limited sampling), published plasma concentration–time profiles following oral dose were searched, digitized and dose-normalized to derive a plasma AUC_0–24h_. Pharmacovigilance data from the U.S. Food and Drug Administration Adverse Event Reporting System (FAERS) database and Eudravigilance were analyzed for “*lorazepam related exposure via human milk*”.

**Results:**

Milk concentrations ranged from 0.65 to 2.10 ng/mL, with highest levels at 1.5 h. The milk AUC_0–24h_ was 25.36 ng*h/mL. Literature-derived plasma AUC_0–24h_ was 158.94 ng*h/mL resulting in an AUC-based M/P ratio of 0.16, calculated DID was 218 ng/kg/day. Assuming milk intakes of 150 and 200 mL/kg/day, estimated DID values were 176.26 and 235.02 ng/kg/day respectively, corresponding to RID values of 1.35% and 1.80%, increasing up to 3.85% upper-bound estimates. The RID_therapeutic_ was 0.44%, increasing to 0.81% in upper-bound estimates. FAERS identified 25 reports of lorazepam exposure via human milk, Eudravigilance one, with mainly central nervous system (CNS) related symptoms. In all these cases, lorazepam was part of CNS polypharmacy, while no lorazepam monotherapy related events were retrieved.

**Conclusion:**

These findings suggest low infant exposure following occasional single, low-dose lorazepam use during breastfeeding. While interpretation should remain cautious given the single-case design, the single low dose, limited plasma sampling, and milk concentrations below the validated lower limit of quantification, the available pharmacokinetic data suggest low estimated infant exposure following occasional low-dose lorazepam use. Pharmacovigilance findings provided contextual information but should not be interpreted as confirmatory evidence of safety.

## 1 Introduction

Anxiety-related symptoms are common after delivery, either new or preexisting. In some women, non-pharmacological interventions are insufficient, so that pharmacological treatment becomes part of their care. Population-based studies indicate that benzodiazepine prescribing continues after childbirth for either postpartum anxiety or pre-existing indications. Prescribing patterns may further shift during periods of increased psychological stress (e.g. COVID-19 pandemic), underscoring the clinical relevance of benzodiazepines in postpartum (Adams et al., 2025; Abraham et al., 2024). A systematic review and meta-analysis reported a pooled prevalence of benzodiazepine use of 0.5% in the year following delivery (Bais et al., 2020). These findings underline that lorazepam use in postpartum is clinically relevant.

Breastfeeding has major health benefits for both the infant and lactating parent. Therefore, avoiding unnecessary discontinuation is an important clinical goal. Beyond individual clinical benefits, breastfeeding is associated with substantial population-level health and economic advantages. Unnecessary discontinuation has been shown to increase healthcare costs and long-term disease burden. (Victora et al., 2016; Rollins et al., 2016; Alive and Thrive, 2025). When medicines with central nervous system (CNS) effects are used in postpartum, concerns about infant exposure via human milk frequently lead to conservative clinical advice. In practice, this may result in avoidable interruption of breastfeeding or “pump-and-dump” strategies despite limited justification (Mitchell et al., 2026). Contemporary recommendations increasingly emphasize individualized, pharmacokinetics-informed decision-making, incorporating maternal dose and dosing pattern, drug half-life and metabolites, and quantitative infant exposure metrics such as the relative infant dose (RID), preferably in combination with pharmacovigilance analyses.

Nevertheless, for benzodiazepines used outside the peri-operative setting remains largely derived from small observational datasets and case reports, leaving uncertainty in routine care, even more after the first week postpartum (Mitchell et al., 2026; NHS Specialist Pharmacy Service). Overall, specific data on lorazepam exposure through human milk remain scarce. Lorazepam is listed as a preferred benzodiazepine during breastfeeding in current UK specialist guidance. This is consistent with its relatively short elimination half-life, absence of major active metabolites, and low reported milk concentrations (Specialist Pharmacy Service, 2024; Drugs and Lactation Database, 2024; Genus Pharmaceuticals, 2026; Anderson, 2022). Published pharmacokinetic studies indicate that peak plasma concentrations after oral lorazepam are typically reached within approximately 2–2.5 h (Greenblatt et al., 1976; 1982).

Observational clinical data on infant outcomes during breastfeeding are limited but informative. In a prospective cohort study of 124 breastfeeding women using benzodiazepines, lorazepam accounted for 52% of exposures during lactation. Reported adverse events in breastfed infants were uncommon, with CNS depression observed in 1.6% of cases, occurring exclusively in the context of chronic maternal use and concomitant exposure to multiple CNS-depressant medications (Kelly et al., 2012). These findings suggest that infant adverse events appear to be more closely related to dosing pattern and polypharmacy than to benzodiazepine exposure *per se*.

Given the ongoing use of benzodiazepines after delivery and the remaining uncertainty surrounding their use during breastfeeding, additional real-world evidence is warranted to support clinical decision-making. In this context, we focused on lorazepam and report a case of infant exposure during breastfeeding, included in the UmbrelLACT study (Van Neste et al., 2024). To contextualize potential infant exposure beyond the individual case, we also explored pharmacovigilance data from the Food and Drug Administration (FDA) Adverse Event Reporting System (FAERS) and the Eudravigilance databases.

## 2 Methods

### 2.1 Study design and ethical considerations

#### 2.1.1 Case report and ethics

This case report describes pharmacokinetic assessment of lorazepam transfer into human milk 6.5 months postpartum after a single intake (*as needed*). Lorazepam treatment was initiated and managed as part of the standard clinical care, independent of study participation. The study team was not involved in the decision to initiate or modify treatment, nor in any other aspect of clinical management.

Human milk and maternal plasma samples were collected over a 24-h period following lorazepam intake to characterize milk and plasma concentration–time profiles and to estimate infant exposure.

This report describes a single lactation pharmacokinetic case nested within the UmbrelLACT study (ClinicalTrials.gov identifier: NCT06042803), the protocol of which has been published previously (Van Neste et al., 2024). The UmbrelLACT study was approved by the Ethics Committee Research UZ/KU Leuven (internal study number S67204). Written informed consent was obtained from the mother for both herself and her infant prior to enrollment. After inclusion, maternal demographic and clinical characteristics, including anthropometry, pregnancy and breastfeeding information, and medication use in the days preceding sample collection, were obtained using standardized self-reporting questionnaires (Van Neste et al., 2024).

#### 2.1.2 Sample collection

Sample collection procedures and documentation were conducted within the framework of the UmbrelLACT study protocol (Van Neste et al., 2024).

Human milk and maternal plasma samples were collected over a 24-h period starting from a single (1 mg) oral lorazepam intake. Milk sampling was performed at home using an electric breast pump. During the 24-h sampling window, the participant was instructed to express all human milk at each expression session. For each expression, the total milk volume from both breasts was recorded. The mother expressed when she would normally feed the infant.

From each expression session, an aliquot of approximately 2–10 mL (not exceeding 10% of the total expressed volume) was transferred into labeled Falcon tubes using a clean syringe. The exact time of milk expression, sample label, and timing of storage were documented for each sample. Immediately after collection, milk samples were stored in the participant’s refrigerator at 4 °C for a maximum of 24 h. All milk samples were subsequently transferred on ice to the study center and stored at −80 °C until bioanalysis.

Maternal venous blood samples were collected on the same sampling day to characterize systemic exposure. One blood sample was obtained within 1 h of the first milk expression following lorazepam intake, and a second blood sample was collected within 1 h of the last milk expression session. Blood samples were transported to the study center on ice, samples were stored at −80 °C until analysis.

#### 2.1.3 Bioanalysis

Bioanalysis was performed on samples collected and stored according to the predefined sampling and storage procedures. Lorazepam was quantified by the laboratory of the Department of Clinical Pharmacy and Pharmacology of the University Medical Center Groningen. Lorazepam was quantified using Ultra-Performance Liquid Chromatography–Tandem Mass Spectrometry (UPLC-MS/MS) after stable isotope dilution according to European Medicines Agency (EMA) guideline (European Medicines Agency, 2022). The analysis was designed and validated for human plasma and serum, and cross-validated for human milk using blanc milk samples. The laboratory is ISO 15189 accredited and participates in proficiency testing for lorazepam with sufficient results.

The lower limit of quantification (LLOQ) was 5 ng/mL. Concentrations below this threshold were considered below the quantifiable range. For pharmacokinetic and infant exposure calculations, two analytical approaches were applied. In the PK analysis, measured or estimated concentrations were used as reported.

#### 2.1.4 Pharmacokinetic and infant exposure calculations

The area under the concentration–time curve over 24 h (AUC_0–24h_) was estimated using the linear trapezoidal method with interpolation to 24 h based on the last observed concentration in human milk.

The average concentration over 24 h (Cav_0–24h_) in human milk was calculated as follows:

Equation 1 *Average Milk Concentration*

$${\text{Cav}}_{0-24h}=\frac{{\text{AUC}}_{0-24h}}{24}$$ (1)

To characterize the relationship between milk and maternal systemic exposure, single time point milk-to-plasma concentration ratios (M/P ratios) were calculated using paired milk and plasma samples when available (Kanto, 1982).

Equation 2 Milk*-to-Plasma Ratio*

$$\frac{M}{P}\text{ratio}=\frac{C_{milk}}{C_{plasma}}$$ (2)

In addition, an AUC-based M/P ratio (both maternal) was estimated to provide an integrated measure of drug transfer into human milk over time.

To contextualize systemic exposure observed in the present maternal case, previously published oral lorazepam pharmacokinetic studies reporting a 2 mg dose and providing sufficient graphical resolution for digitization of mean plasma concentration–time profıles were retrieved and used (Kamal et al., 2010). Plasma concentration–time data were digitized from published figures using WebPlotDigitizer (version 5, Automeris LLC, USA). Digitized data points were used to reconstruct concentration–time curves. Plasma AUC_0–24h_ from the literature-derived profiles was calculated using the linear trapezoidal method with interpolation to define the 24-h boundary. Due to limited plasma sampling (n = 2), reliable reconstruction of a full plasma concentration–time curve in the present case was not feasible. Therefore, literature-derived pharmacokinetic profiles were used to estimate systemic exposure. However and for comparison, plasma AUC_0–24h_ in the present case was approximated using the available plasma samples and linear trapezoidal interpolation, acknowledging the limitations of this approach.

Dose normalization was applied to enable comparison across doses by expressing AUC_0–24h_ per milligram of administered dose. Specifically, AUC_0–24h_ values derived from digitized 2 mg mean concentration–time profiles were divided by the administered dose to obtain 1 mg-normalized exposure estimates. This approach assumes approximate dose proportionality (‘linear’) over the therapeutic range, as reported in adult pharmacokinetic studies. Single-dose studies in healthy volunteers have shown that plasma lorazepam concentrations increase proportionally with dose (Shader et al., 1986). Additional pharmacokinetic literature similarly describes dose-independent clearance and proportional exposure within the standard therapeutic dose range (Greenblatt et al., 1979).

Under these conditions of established dose proportionality, AUC values derived from 2 mg oral dosing were divided by two to obtain exposure estimates corresponding to a 1 mg oral dose. These dose-normalized plasma AUC_0–24h_ values were then compared with milk AUC_0–24h_ to calculate the AUC-based milk-to-plasma ratio.

Equation 3 *Dose Normalization*

$$\text{AUC normalized}=\frac{{\text{AUC}\;}_{\text{observed}}}{\;\text{Dose}}$$ (3)

Equation 4 *Dose-normalized AUC*_*0–24h*_ *(1 mg)*

$${AUC}_{0-24h}\left(1\text{ mg}\right)=\frac{{\text{AUC}\;}_{0-24h}\left(2\;mg\right)}{2}$$ (4)

Equation 5 *AUC-based Milk-to-Plasma Ratio*

$$\frac{M}{P}\text{ratio}\left(\text{AUC}-\text{based}\right)=\frac{AUC_{\;milk}}{AUC_{plasma}}$$ (5)

These complementary approaches allow assessment of both point-specific and time-integrated milk-to-plasma relationships.

Infant exposure was estimated by calculating the DID and RID. The DID was calculated using two approaches: first, from individual milk sample data together with the milk volumes collected in this specific case; and second, from the calculated average milk concentration using standardized milk intake assumptions of 150 and 200 mL/kg/day to allow comparison across different scenarios.

Equation 6 *Daily Infant Dosage in this reported case*

$$DID\;\left(\frac{\frac{ng}{kg}}{day}\right)=\sum_{i=1}^{n}\left(\frac{Milk\;Concentration_{i}\left(\frac{ng}{mL}\right)*Milk\;Volume_{i}\left(mL\right)}{Infant\;weight\;\left(kg\right)}\right)$$ (6)

Equation 7 *Daily Infant Dosage as a function of standardized milk intake*

$$DID\;\left(\frac{\frac{ng}{kg}}{day}\right)=\text{Average Milk Concentration }\left(\text{Cav},0-24\;h\right)\;\left(\frac{ng}{mL}\right)$$ (7)

$$*Infant\;Milk\;Intake\;\left(\frac{\frac{mL}{kg}}{day}\right)$$

The (RID) was calculated by dividing the estimated DID (ng/kg/day) by the maternal weight-normalized daily dose (ng/kg/day) on the sampling day and expressing the result as a percentage.

Equation 8 *Relative Infant Dose*

$$RID\;\left(\%\right)=\frac{DID\;\left(\frac{\frac{ng}{kg}}{day}\right)}{Daily\;Maternal\;Dose\;\left(\frac{ng}{day}\right)/\;Maternal\;Weight\;\left(kg\right)}*100$$ (8)

In addition to RID, a relative infant therapeutic dose (RID_therapeutic_) was calculated to contextualize infant exposure relative to a reference therapeutic dose used in infants. The reference infant therapeutic dose was derived from published pediatric oral lorazepam dosing recommendations, using the lower end of the reported dose range for children under 2 years of age (25 μg/kg twice daily), corresponding to 50,000 ng/kg/day (PatchSA, 2026).

Equation 9 *Relative Infant Therapeutic Dose*

$$RID_{therapeutic}\left(\%\right)=\frac{DID\;\left(\frac{\frac{ng}{kg}}{day}\right)}{Daily\;Therapeutic\;Infant\;Dosage\;\left(\frac{\frac{ng}{kg}}{day}\right)}*100$$ (9)

The RID_therapeutic_ was calculated from the estimated DID values obtained under each exposure scenario and expressed as a percentage of a reference infant therapeutic dose. This calculation was applied consistently to DID values derived from both patient-specific milk volumes and standardized milk intake assumptions.

For concentrations below the LLOQ, infant exposure and pharmacokinetic parameters were calculated using two approaches: (1) a primary analysis using estimated concentrations as reported, and (2) an upper-bound scenario substituting concentrations below the LLOQ with LLOQ/2, providing conservative upper-bound exposure estimates, a conventional substitution approach (Beal method M5) commonly applied in pharmacokinetic analyses (Beal, 2001). All pharmacokinetic calculations and graphical representations were performed using Microsoft Excel 2016 (Microsoft Corporation, Redmond, WA, USA).

### 2.2 Pharmacovigilance dataset exploration

#### 2.2.1 The FDA Adverse Event Reporting System

All publicly available FAERS (Silver spring, MD, USA) records were queried on 18 February 2026. FAERS collects spontaneous adverse event (AE) reports submitted by healthcare professionals, consumers, and manufacturers worldwide. Duplicate and follow-up entries were consolidated according to FDA guidance, retaining the most recent version for analysis.

Breastfeeding-related reports were identified and subsequently filtered according to the following exposure routes: (a) exposure via human milk, (b) breastfeeding, (c) intoxication by breastfeeding, or (d) maternal exposure during breastfeeding, similar to an earlier analysis (Tezel Yalcin et al., 2024). This list was subsequently screened for lorazepam. For the infant population under study, a maximum weight limit was set at 15 kg. Individual case listings were reviewed descriptively, with aspects related to year and country of reporting, age, sex, seriousness, or co-medication, with specific focus on CNS-relevant medicines and adverse events.

Disproportionality analyses were conducted using Reporting Odds Ratio (ROR), Proportional Reporting Ratio (PRR), and Information Component (IC) methods. For each adverse event of interest (CNS focused), 2 × 2 contingency tables were constructed comparing infant lorazepam reports within the breastfeeding subset of FAERS with all other breastfeeding-related reports. Signal detection was considered when the lower bound of the 95% confidence interval of the ROR exceeded 1, PRR was ≥2, and IC025 (lower 95% credibility interval of IC) was greater than 0.

#### 2.2.2 Eudravigilance

The EudraVigilance database was explored to complement the FAERS analysis. The database was queried for reports involving “*lorazepam*” over an extended period from 1994 to 22 March 2026, with the MedDRA preferred term “*Exposure via breastmilk*”, further building on the recent analysis on suspected adverse drug reactions in infants through breastfeeding (Heerforst et al., 2025).

Reports were filtered for cases involving infants aged 0–2 years. Data were categorized by reporting time, infant age and sex, seriousness and type of adverse drug reaction, and the medications involved with particular attention to CNS active drugs. Individual case narratives were reviewed when available.

Analyses were descriptive in nature and not intended to estimate incidence or establish causality.

## 3 Results

### 3.1 Case characteristics

A breastfeeding mother in postpartum (day 202–203 after delivery) received an occasional (*as needed)* single 1 mg oral dose of lorazepam (time 0) on 17 September 2024 (14:00) as part of her clinical care (*as needed* use of lorazepam for anxiety-related symptoms). Lorazepam therapy had been initiated 15 months earlier, and was used intermittently for symptomatic control. Maternal body weight on the sampling day was 77 kg, the breastfed infant weighed 6.055 kg. Concomitant products included a gamma-aminobutyric acid (GABA) supplement (Deba Pharma GABA 500 mg once daily; initiated 1 July 2024), a thyroid support supplement (Thyrotabs®, once daily; initiated 15 July 2024), and diclofenac retard 75 mg once daily (initiated 10 June 2024). Human milk samples (n = 6) and maternal plasma samples (n = 2) were collected over a 24-h period following lorazepam intake to characterize milk and plasma concentration–time profiles and to estimate infant exposure. No adverse events were reported in either the mother or the breastfed infant.

### 3.2 Maternal plasma concentrations and systemic exposure

Two maternal plasma samples were obtained 2.1 and 23.6 h after lorazepam intake. Measured plasma concentrations were 10.77 ng/mL and 3.69 ng/mL, respectively, with the latter being below the LLOQ (5 ng/mL).

### 3.3 Human milk concentration–time profile

**FIGURE 1:**
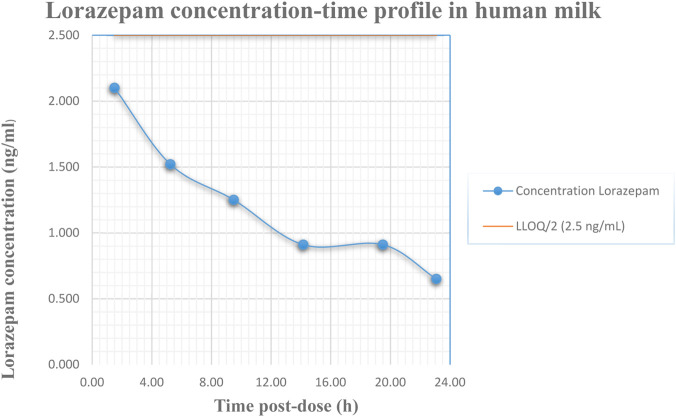
The human milk concentration–time profile for 24 h following oral intake of 1 mg of lorazepam (LLOQ = lower limit of quantification was 5 ng/mL, so that LLOQ/2 was 2.5 ng/mL). All measured milk concentrations were below the validated LLOQ of 5 ng/mL and are therefore reported as semi-quantitative values. The horizontal line indicates the LLOQ/2 substitution value (2.5 ng/mL) used for the upper-bound exposure scenario.

Human milk samples (n = 6) were collected between 1.5 and 23.1 h after lorazepam intake. Measured human milk concentrations ranged from 0.65 to 2.10 ng/mL, with the highest observed human milk concentration (C_max_) at 1.5 h post-dose (2.10 ng/mL). Concentrations declined over time to 0.65 ng/mL at 23.1 h (Figure 1). All measured milk concentrations were below the LLOQ (5 ng/mL) and are therefore reported as semi-quantitative values. Accordingly, all pharmacokinetic estimates derived from these milk concentrations should be considered exploratory and interpreted with appropriate caution.

Using linear trapezoidal integration without extrapolation beyond the last observed concentration, the milk AUC_0–24h_ was 25.36 ng*h/mL. The corresponding average milk concentration over 24 h (C_av,0–24h_) was 1.175 ng/mL. In an upper bound scenario applying LLOQ/2 substitution for concentrations below the quantification limit, the estimated milk AUC_0–24h_ was 53.96 ng*h/mL, The corresponding mean concentration over the observed sampling interval (1.5–23.1 h) was 2.5 ng/mL.

### 3.4 Milk-to-plasma ratios

Milk-to-plasma (M/P) ratios were calculated using temporally paired milk and plasma samples. Using measured concentrations, the M/P ratio was 0.1949 at 2.1 h (M1/P1) and 0.1761 at 23.6 h (M6/P2), yielding a mean M/P ratio of 0.1855. In upper-bound scenario applying LLOQ/2 substitution for concentrations below LLOQ, the corresponding M/P ratios were 0.2321 and 1.000, with a mean M/P ratio of 0.6160.

Since single time point M/P ratios may be misleading, as milk and plasma concentrations do not necessarily change in parallel over time (Anderson, 2025), an AUC-based M/P ratio was also calculated to provide a more robust assessment of drug transfer into human milk. Mean plasma concentration–time data from Kamal et al. (2010) following a 2 mg oral dose were digitized to derive a literature-based plasma AUC_0–24h_ of 317.89 ng*h/mL. After dose normalization, the corresponding plasma AUC_0–24h_ for a 1 mg dose was 158.94 ng*h/mL.

Using this literature-derived plasma AUC_0–24h_, the AUC-based M/P ratio was calculated by comparing it with the milk AUC0-24 h (25.36 ng*h/mL) yielding a value of 0.16. For comparison, using the same methodological approach based on the two maternal plasma samples in the present case, the estimated plasma AUC_0–24h_ was 155.69 ng*h/mL, resulting in a similar AUC-based M/P ratio of 0.16.

### 3.5 Infant exposure

Infant exposure was estimated using two approaches: (1) using patient-specific milk volumes collected in the present case, and (2) based on standardized milk intake assumptions.

Based on the actual collected milk volumes over the 24-h sampling period, the patient-specific DID was 218 ng/kg/day. Applying standardized milk intake of 150 mL/kg/day, the estimated DID was 176.26 ng/kg/day. Under an intake assumption of 200 mL/kg/day (early infancy scenario), the DID was 235.02 ng/kg/day.

In an upper-bound scenario applying LLOQ/2 substitution for concentrations below the limit of quantification, the estimated DID increased to 404.62 ng/kg/day for the patient-specific approach, and to 375 and 500 ng/kg/day under average and early infancy intake assumptions, respectively.

The corresponding relative infant dose (RID) for the patient-specific approach was 1.68%, increasing to 3.11% under LLOQ/2 substitution. Under standardized intake assumptions, RID values were 1.35% and 1.80% for average and early infancy scenarios, respectively, and increased to 2.88% and 3.85% in the upper-bound scenario.

Similarly, the RID_therapeutic_ for the patient-specific approach was 0.44%, increasing to 0.81% under LLOQ/2 substitution. Under standardized intake assumptions, RID_therapeutic_ values were 0.35% and 0.47% for average and early infancy scenarios, respectively, and increased to 0.75% and 1.00% when applying LLOQ/2 substitution. No adverse events were reported.

### 3.6 Pharmacovigilance findings

#### 3.6.1 FAERS

In total, 12,527 breastfeeding related events were identified. Lorazepam exposure was reported in only 25 cases. The age ranged from 1 week until 17 months (one case not specified). There were nine female, and 11 males infants (5 not specified). The countries involved were United States (n = 10), Germany (n = 8), Great Britain (n = 5), Finland (n = 1) and Italy (n = 1). All were classified as serious.

None of the events was reported after mono-therapy with lorazepam, and most commonly reported co-medications included opioids (fentanyl, morphine, oxycodone), other benzodiazepines (clobazam, lorazepam), anti-epileptic drugs (levetiracetam, topiramate) or antidepressants (sertraline). Disproportionality analyses were conducted for 11 specific adverse neurological events of interest, using ROR, PRR, and IC in Table 1. These events mainly reflect CNS related events (Table 2).

**TABLE 1:** Human milk lorazepam concentrations following a single 1 mg oral dose.

Time post-dose (h)	Human milk lorazepam concentration (ng/mL)	Quantification status
1.5	2.10	< LLOQ; semi-quantitative
5.25	1.52	< LLOQ; semi-quantitative
9.5	1.25	< LLOQ; semi-quantitative
14.16	0.91	< LLOQ; semi-quantitative
19.5	0.91	< LLOQ; semi-quantitative
23.1	0.65	< LLOQ; semi-quantitative

LLOQ, 5 ng/mL. All concentrations were below the validated LLOQ, and should therefore be considered semi-quantitative.

**TABLE 2:** Exploratory disproportionality analysis of lorazepam related events, weighted to all breastfeeding related events as retrieved in the FAERS dataset.

Adverse event	n	ROR (95% CI)	PRR	IC	IC_025_
Somnolence	11	33.6 (15.1–74.0)	19.2	4.21	3.62
Lethargy	10	69.4 (30.6–157.9)	42.0	5.28	4.66
Respiratory depression	10	193.2 (82.2–450.6)	116.3	6.56	5.94
Agitation	10	69.4 (30.6–157.9)	42.0	5.28	4.66
Confusional state	10	259.8 (111–608)	156.4	6.90	6.28
Tachycardia	10	116.8 (50.0–273.0)	70.6	6.04	5.42
Hyperglycaemia	10	462.4 (190–1,124)	277.9	7.66	7.04
Irritability	9	33.4 (14.6–76.5)	20.8	4.30	3.65
Acidosis	9	109.3 (46.6–256.2)	66.4	5.95	5.30
Apnoea	8	42.2 (17.8–99.5)	29.1	4.78	4.09
Hypopnoea	8	840.8 (273–2,570)	571.0	8.06	7.37

Abbreviations: ROR, reporting odds ratio; PRR, proportional reporting ratio; IC, information component; IC025, lower limit of the 95% confidence interval of the IC.

#### 3.6.2 Eudravigilance

A search of the EudraVigilance database for lorazepam-related reports over the period 1994 to 22 March 2026 identified a total of 20,381 individual cases. Of these, 309 cases involved children aged 0–2 years (154 = aged 0–1 month, and 155 = aged 2 months to 2 years). Among all identified reports, only one case was coded with the MedDRA preferred term “Exposure via human milk.” In this case, reported adverse reactions included neonatal somnolence, increased drug levels and drug exposure via human milk. The lorazepam dose was 1 mg/day, co-reported medications included different CNS-active (olanzapine, and citalopram) agents.

## 4 Discussion

The present case report provides pharmacokinetic characterization of lorazepam transfer into human milk following a single oral dose (1 mg) administered to a breastfeeding mother 6.5 months postpartum.

An important analytical limitation is that all measured milk concentrations were below the validated lower limit of quantification (LLOQ). Although these concentrations demonstrated a consistent temporal decline and were therefore retained for exploratory pharmacokinetic assessment, they should be regarded as semi-quantitative estimates. Consequently, the calculated AUC, M/P ratios, and infant exposure estimates should be interpreted as approximate rather than definitive pharmacokinetic measures.

Overall, lorazepam concentrations in human milk samples were low. The highest observed milk concentration was 2.10 ng/mL at 1.5 h after dosing, and the average milk concentration over 24 h was 1.18 ng/mL, corresponding to a milk AUC_0–24h_ of 25.36 ng*h/mL. Estimated infant exposure remained limited, with a calculated RID ranging from 1.35% to 1.80% under standard milk intake assumptions and up to 3.85% in the upper-bound scenario. These values are well below the commonly accepted 10% threshold of the maternal weight-adjusted dose, which is widely used as a pragmatic benchmark for breastfeeding compatibility (Begg et al., 2002; Drugs and Lactation Database, 2024; Rowland Yeo et al., 2025). The M/P ratios (either paired, or based on AUC_0–24h_) were consistently low, indicating limited transfer of lorazepam into human milk. Published lactation data are concordant with this interpretation, reporting low lorazepam concentrations in milk and low milk-to-plasma ratios in lactating women, while protein binding and lipophilicity are recognized more generally as determinants of drug partitioning into milk (Nishimura et al., 2021; National Library of Medicine, 2006; Hale, 2021). Lorazepam is substantially bound to plasma proteins (approximately 85%), which may further limit its transfer into breast milk (Valeant Pharmaceuticals North America LLC, 2016). Maternal systemic exposure following the 1 mg oral dose was comparable to dose-normalized exposures reported in previous pharmacokinetic studies, supporting the internal consistency of the observed concentration–time profiles. The first plasma sample was collected at 2.1 h post-dose, which falls within the expected time to peak plasma concentration reported after oral lorazepam administration (approximately 2–2.5 h) (Greenblatt et al., 1976; 1982), although the limited sampling precludes precise determination of plasma Cmax. The systemic exposure in the present postpartum case was comparable to the exposure reported in non-pregnant adults (Kamal et al., 2010), while higher than values reported during pregnancy/at labor (Papini et al., 2006). This reflects the altered pharmacokinetics during pregnancy (increased liver metabolism and clearance) and a return toward non-pregnant exposure patterns in the postpartum period. The literature-derived plasma AUC_0–24h_ was not intended to replace individual pharmacokinetic measurements but rather to provide a pragmatic exploratory reference for contextualizing milk exposure in the setting of *a priori* intended sparse plasma sampling.

Lorazepam is known to exhibit largely linear pharmacokinetics, with similar systemic exposure observed after single and multiple dosing, supporting the predictability of dose-normalized exposure comparisons with previously published pharmacokinetic studies, be it with some accumulation towards steady state during repeated dosing (Chaudhary et al., 1993).

The estimated AUC_0–24h_ M/P ratio in the present case was 0.16 relative to maternal systemic concentrations. These findings are consistent with previously reported lactation data, where milk concentrations of approximately 8–9 ng/mL and corresponding M/P ratios of 0.15–0.26 have been described (Sommerfield and Nielsen, 1985), as well as RID around 2%–3% as reported in lactation references (Hale, Medications and Mothers’ Milk). Consistent with this interpretation, drug safety databases also report that lorazepam is present in human milk in low concentrations and that the amount ingested by the nursing infant is typically pharmacologically insignificant (International Business Machines Micromedex, 2026; Lorazepam, 2026).

Based on the measured milk concentrations, the calculated RID ranged between approximately 1.36% and 1.81% of the maternal weight-adjusted dose under standard assumptions, and up to 3.85% in upper-bound scenario. These findings are consistent with previously reported data on benzodiazepine transfer into human milk. In a study evaluating several benzodiazepines in lactating women, the reported M/P ratio for lorazepam was approximately 0.21–0.26, and the RID was around 2.1%–4.4% (Nishimura et al., 2021). Along the same line, the RID_therapeutic_ was also very low. This is consistent with a low likelihood of clinically relevant pharmacological effects in the infant, when applying NOAEL concepts to the RID_therapeutic_ (<1%) findings.

These pharmacokinetic findings are consistent with clinical observations suggesting a low incidence of adverse infant outcomes following benzodiazepine exposure as monotherapy during breastfeeding in cohort studies (Kelly et al., 2012). Previous clinical observations have likewise suggested that benzodiazepine exposure during breastfeeding is generally associated with a low incidence of adverse infant outcomes when maternal dosing is limited and not combined with other CNS depressants (Iqbal et al., 2002; Kelly et al., 2012).

To further contextualize the clinical relevance of these pharmacokinetic case findings, pharmacovigilance data from both the FAERS and EudraVigilance databases were considered and explored. These findings should be interpreted as hypothesis-generating only, given the limited number of lorazepam reports and frequent CNS polypharmacy. Taking this into account, CNS-active drugs were among the most frequently co-reported medication classes in breastfeeding-related adverse event reports.

The number of cases specifically involving lorazepam in polypharmacy remained limited, and absent in monotherapy. In the FAERS database, we retrieved 25 serious adverse events and only one case was identified in the Eudravigilance database. Importantly, no pharmacovigilance cases involving lorazepam monotherapy were retrieved. In our previous study evaluating all medications used by breastfeeding mothers, adverse events in infants where lorazepam was part of a combination therapy were reported at a rate of 0.91% (Tezel Yalcin et al., 2024). In the current study, this rate was found to be 0.2%. This finding indicates that, within the FAERS database, adverse events associated with monotherapy are reported at a significantly lower frequency compared to combination therapies. Taken together, the limited number of reports in both FAERS and EudraVigilance are consistent with infrequent reporting of adverse effects following lorazepam exposure during breastfeeding. Obviously, underreporting and confounding by concomitant medications preclude any robust conclusions regarding incidence or safety, since, e.g., underreporting is a well-recognized limitation of spontaneous pharmacovigilance systems and may substantially underestimate the true frequency of adverse events (Byskov et al., 2024).

While there is value in the case report and the pharmacovigilance analysis, several limitations should be acknowledged. First, this report represents a single clinical case, with specific characteristics (1 mg, single dose) which limits generalizability. Second, plasma sampling in the present case was sparse, restricting the precision of maternal pharmacokinetic parameter estimation and the need to use AUC_0–24h_ plasma estimates from literature. The use of previously published pharmacokinetic studies relied on digitization of published concentration time curves, which may introduce minor estimation errors. Furthermore and be it in line with the study protocol, questionnaires were based on self-reporting, while no blood sampling in the infant was performed. Finally, underreporting or biases are inherent to any pharmacovigilance database analysis.

In this single breastfeeding case involving an occasional 1 mg oral lorazepam dose administered to a mother nursing a healthy 6.5-month-old infant, milk transfer and estimated infant exposure were low. These findings should not be extrapolated to repeated dosing, higher maternal doses, neonates, premature infants, medically fragile infants, or situations involving concomitant sedating medications; pharmacovigilance data did not identify a clear signal for uncomplicated monotherapy, although these findings remain exploratory and should not be used to infer incidence.

In conclusion, the concordance between low pharmacokinetic exposure (low AUC, low M/P ratio and low RID_therapeutic_ (<1%)) and the limited pharmacovigilance reporting on monotherapy should be viewed as contextual rather than confirmatory evidence. Taken together, the available data suggest that occasional low-dose lorazepam use during breastfeeding is associated with low estimated infant exposure. Additional lactation pharmacokinetic and pharmacovigilance studies are needed to further refine and confirm these observations.

## Data Availability

The original contributions presented in the study are included in the article/supplementary material, further inquiries can be directed to the corresponding author.
